# Estimating the share of SARS-CoV-2-immunologically naïve individuals in Germany up to June 2022

**DOI:** 10.1017/S0950268823000195

**Published:** 2023-02-15

**Authors:** Benjamin F. Maier, Annika H. Rose, Angelique Burdinski, Pascal Klamser, Hannelore Neuhauser, Ole Wichmann, Lars Schaade, Lothar H. Wieler, Dirk Brockmann

**Affiliations:** 1 Robert Koch Institute, Berlin, Germany; 2 DTU Compute, Technical University of Denmark, Kongens Lyngby, Denmark; 3 Copenhagen Center for Social Data Science, University of Copenhagen, Copenhagen, Denmark; 4 Institute for Theoretical Biology and Integrated Research Institute for the Life-Sciences, Humboldt University of Berlin, Berlin, Germany

**Keywords:** COVID-19, immunity, modelling

## Abstract

After the winter of 2021/2022, the coronavirus disease 2019 (COVID-19) pandemic had reached a phase where a considerable number of people in Germany have been either infected with a severe acute respiratory syndrome coronavirus 2 (SARS-CoV-2) variant, vaccinated or both, the full extent of which was difficult to estimate, however, because infection counts suffer from under-reporting, and the overlap between the vaccinated and recovered subpopulations is unknown. Yet, reliable estimates regarding population-wide susceptibility were of considerable interest: Since both previous infection and vaccination reduce the risk of severe disease, a low share of immunologically naïve individuals lowers the probability of further severe outbreaks, given that emerging variants do not escape the acquired susceptibility reduction. Here, we estimate the share of immunologically naïve individuals by age group for each of the sixteen German federal states by integrating an infectious-disease model based on weekly incidences of SARS-CoV-2 infections in the national surveillance system and vaccine uptake, as well as assumptions regarding under-ascertainment. We estimate a median share of 5.6% of individuals in the German population have neither been in contact with vaccine nor any variant up to 31 May 2022 (quartile range [2.5%–8.5%]). For the adult population at higher risk of severe disease, this figure is reduced to 3.8% [1.6%–5.9%] for ages 18–59 and 2.1% [1.0%–3.4%] for ages 60 and above. However, estimates vary between German states mostly due to heterogeneous vaccine uptake. Excluding Omicron infections from the analysis, 16.3% [14.1%–17.9%] of the population in Germany, across all ages, are estimated to be immunologically naïve, highlighting the large impact the first two Omicron waves had until the beginning of summer in 2022. The method developed here might be useful for similar estimations in other countries or future outbreaks of other infectious diseases.

## Introduction

The coronavirus disease 2019 (COVID-19) pandemic caused by the rapid global dissemination of severe acute respiratory syndrome coronavirus 2 (SARS-CoV-2) and its respective variants has led to a large number of infections worldwide [1]. In Germany, around 21.4 million infections had been reported until the end of May 2022. Moreover, a large part of the population had received a primary vaccination series with one of the available COVID-19 vaccines (mRNA-vaccine by BioNTech or Moderna or a vector-based vaccine by AstraZeneca or Janssen) at the time [2]. The national COVID-19 vaccination campaign began at the end of 2020 by targeting older adults, residents of nursing homes, and healthcare workers, then shifting focus to younger adults [3]. In August 2021, a recommendation to vaccinate adolescents aged 12–17 was issued and since December 2021, children aged 5–11 years were recommended to receive a vaccination if underlying medical conditions put them at increased risk for severe disease [4, 5]. In Germany, recovered individuals were advised not to receive a COVID-19 vaccination until 6 months [6] or 3 months [7] have passed after infection, respectively. At the time of analysis, booster vaccinations had been recommended for all persons aged 11 years and older [8, 9]. A central factor that would determine how the pandemic progressed in Germany was the number of people still immunologically naïve to infection, i.e. that have neither been in contact with the virus or any of its variants nor a vaccine against them. In Germany, several serological studies have been conducted [10, 11] but up until spring 2022 no data that extended into the time of the Omicron waves was available, particularly with respect to children [12]. Therefore, at the time we chose a mathematical modelling approach to estimate the number of immunologically naïve individuals in order to facilitate informed decisions with regard to the future trajectory of the pandemic in Germany.

To estimate the number of people that have been in contact with either virus or vaccine, one might simply summate the number of vaccinations and the number of reported infections. However, doing so ignores the fact that (a) a considerable number of vaccinated people have suffered from additional breakthrough infections (taking into account both asymptomatic and symptomatic infections herein) [13], (b) a substantial number of previously infected people have chosen to be vaccinated in accordance with national recommendations [14–16], (c) some individuals have suffered from multiple infections [17] and (d) the exact extent of the total number of infections as compared to the reported number of infections is unknown because (i) asymptomatic infections are less likely to be identified and reported in the national surveillance system and (ii) under-ascertainment varies regionally [18, 19]. In order to estimate the overlap between the vaccinated and recovered subpopulations, one may assume that the probability of any recovered individual to be vaccinated is proportional to the probability of any individual to be vaccinated. However, this largely ignores (i) the heterogeneous dynamics of the spreading disease as well as vaccination campaigns and (ii) that vaccinated individuals are less likely to suffer from an infection than unvaccinated individuals [20]. Here, we introduce modelling approaches that are devised to meet the aforementioned conditions and use them to estimate the distribution of immunologically naïve, (in the infectious-disease modelling context called ‘fully susceptible’ hereafter), recovered, and vaccinated individuals in Germany, taking into account regional and age differences. We find that although the percentage of the adult population in Germany that remained fully susceptible was expected to be in the single digits after 31 May 2022, the share of unaffected children might have been considerably larger. Due to heterogeneities in vaccine uptake across German states, these values may differ by region. Our analysis cannot answer questions regarding the quality of achieved immunity against infection or disease, because we consider neither waning of immunity nor the emergence of variants with immune evasive properties, which is difficult to predict [21].

## Methods

We partition the population into *n*_*G*_ = 16 regions corresponding to the German states and *n*_*A*_ = 5 age groups corresponding to ages ‘00–04’ (infants), ‘05–11’ (children), ‘12–17’ (adolescents), ‘18–59’ (adults), ‘60 + ’ (elderly), chosen in accordance with the population structure of publicly available vaccination data [2], i.e. into 80 subpopulations. To obtain nation-wide counts of individuals in age groups, we sum the respective results over all regions, to obtain counts of individuals for all ages, we sum over all age groups. To obtain an age-independent, nation-wide result, we sum over all ages and all regions.

As we are, first and foremost, interested in estimating the proportion of individuals *S*_∞_ ≡ *S*(*t* = *t*_max_) that can be considered to be fully susceptible towards infection with any SARS-CoV-2 variant per region and age group, we report a simplified model here that captures the main ideas and gives the same results for *S*(*t*) as the full model which is reported in the SM, Sec. ‘Introduction’.

We consider the population of size *N* (an age group in a region) to be composed of susceptible (*S*), infected/recovered (*I*), infected/recovered but eligible for reinfection or vaccination (*Y*), vaccinated (*V*) and boostered (*B*) individuals, assuming that the population count is constant over two years such that *N* = *S* + *I* + *Y* + *V* + *B* = const.

The central problem of estimating *S*_∞_ is to determine the overlap between recovered and vaccinated subpopulations. Given that the cumulative number of unvaccinated infected *R*_∞_ and the number of cumulative vaccinated individuals *V*_∞_ is known, one may naïvely assume that the probability that an infected person that was initially unvaccinated is vaccinated later on is proportional to the probability that any person in the population is vaccinated, which is given as *p* = *V*_∞_/*N*. This is in agreement with results of a representative survey study that suggested that recovered individuals had the same intention to vaccinate as individuals that had not yet suffered from an infection [14]. Based on this assumption, the cohort size of unvaccinated and not yet infected individuals would evaluate to *S*_∞_ = *N* − (1 − *V*_∞_/*N*)*R*_∞_ − *V*_∞_. However, doing so largely ignores the time course of infections and vaccinations, with incidence and daily vaccinations peaking at different time points, where a large number of infections occurred after the peak in weekly administered vaccines. Hence, one may assume instead that when a person becomes vaccinated at time *t*, the probability that this person was already infected is proportional to the number of infected/recovered individuals at time *t* that are eligible for vaccination as *p* = *Y*/(*S* + *Y*). With incidence rates of 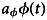 (new unvaccinated cases per day) and vaccination rates of 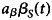 (new vaccinations per day) obtained from data, we assume that the count of individuals in the respective states evolves dynamically as1
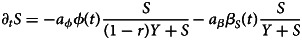
2
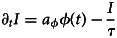
3
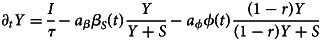
4
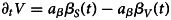
5
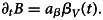


**Fig. 1.**
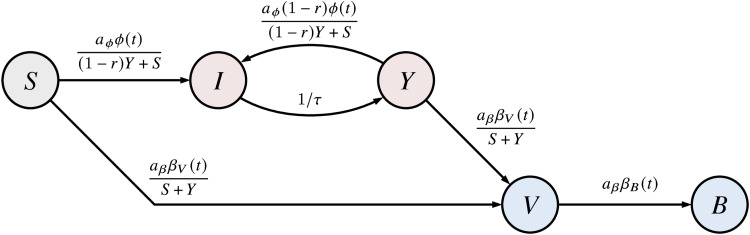
Simplified model schema. On each day, 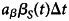 unvaccinated people become vaccinated, with under-ascertainment ratio 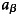 and 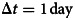. The probability that a newly vaccinated person has been infected before is proportional to the respective size of the subpopulation of recovered people that are eligible for vaccination *Y*. Furthermore, on each day, 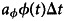 unvaccinated people become infected, with under-ascertainment ratio 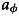. The probability that a newly infected person has been infected before is proportional to the respective size of the subpopulation of recovered people that are eligible for reinfection (1 − *r*)*Y*, where 1 − *r* is the relative reinfection probability or ‘recovered immunity’. Recovered individuals are expected to reach eligibility for reinfection/vaccination after an average duration of *τ*. (Note that in the full model, breakthrough and reinfections of vaccinated individuals are possible (see SM, Sec. ‘Introduction’)).

The last two equations are shown here for completeness, but note that the number of vaccinated and boostered individuals can simply be obtained from data, without integrating the dynamic equations, as their integrals can be evaluated analytically and are equal to the cumulative number of respective vaccinations. Above, 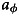 and 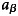 are under-ascertainment ratios that account for infections and vaccinations that have not been reported. The time scale *τ* is equal to the average time after which an infected/recovered individual becomes eligible for reinfection or vaccination and 1 − *r* is the relative probability that an unvaccinated recovered person is reinfected as compared to a fully susceptible individual (Fig. 1).

For our analysis, we draw 1000 pairs of 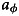 and 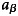 from shifted Gamma distributions with means 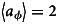, 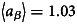 and standard deviations 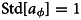, 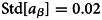 that are bounded below by 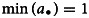. Note that this distribution yields a median under-ascertainment ratio of 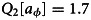, which is in line with results informed by seroprevalence data for Germany in 2020, published as a preprint [19]. Furthermore, with a 97.5th percentile of 4.7, the distribution is broad enough to account for occasional high under-ascertainment ratios that have been observed locally [10, 18, 19]. For infants, ascertainment is expected to be lower than for other age groups [22], which is why we double under-ascertainment ratios for this age group. We did not assume a higher under-ascertainment ratio for children older than 4 years, because regular screening via rapid antigen tests was mandatory in schools across the country for a considerable amount of time [23]. We choose an eligibility time of 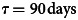, which is approximately of the same order as the time for antibody concentrations to decay after an infection [24]. While it falls in the lower bound of officially recommended time for recovered individuals to wait before getting vaccinated, surveys indicate that people might not strictly follow the official recommendation but get vaccinated earlier. Further, people with asymptomatic courses might have no knowledge about their infection, likely leading to a bias towards shorter times between infection and vaccination in those cases. The influence of lower and higher values of *τ* is investigated in a sensitivity analysis (see SM, Sec. ‘Methods’). The ‘recovered immunity’ parameter *r* quantifies the relative efficacy against reinfection. For the Alpha variant, this efficacy was observed to be lower than the vaccine efficacy against infection by mRNA- or vector-vaccines [25], but of similar order as the vaccine efficacy against infection with Delta, taking on values of *r* ≈ 0.65 for both. As Omicron is considered to be a variant with partial immune escape, we set a lower default value of *r* = 1/2 for all variants, testing *r* = 0 (no protection against reinfection) and *r* = 1 (full immunity) in sensitivity analyses.

Note that we only consider data up to 31 May 2022. We cannot reliably extrapolate the above-listed assumptions regarding under-ascertainment into the summer of 2022 and beyond because the Omicron variant and an increasing population immunity may have altered subjective perception of disease severity and resulting test usage [26].

The daily vaccination rates 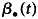 are obtained from data [2] and averaged over calendar weeks to remove weekly modulations. Likewise, infection rates of unvaccinated individuals *ϕ*(*t*) are obtained from reported data in the German reporting system SurvStat [27], which is available in aggregated form upon request. While the vaccination status is unknown for a substantial number of infections, we assume that for every day, the proportion of cases with unknown vaccination status that are, in fact, unvaccinated, is equal to the proportion of unvaccinated cases over the last seven days for which the vaccination status is known. This imputation method is performed for age- and region-stratified data.

For analyses disregarding infections with Omicron, we obtained the nation-wide and age-independent share of randomly sequenced samples in Germany [28] that the software framework ‘scorpio’ identified as ‘Omicron’ or ‘Probable Omicron’ on a per-calendar-week basis by date of extraction (‘Entnahmedatum’) as *σ*(*t*), assuming *σ*(*t*) = 0 for dates previous to 1 August 2021 and *σ*(*t*) = 1 for dates that exceed the last available date in the data. Then, all incidence rates were scaled as *ϕ*_*S*,pre-Omicron_(*t*) = *ϕ*_*S*_(*t*)[1 − *σ*(*t*)]. Note that vaccination rates are unaffected by this procedure.

Population sizes stratified by age and state were requested from destatis [29].

Eqs. (1)–(5) are integrated using Euler's method with 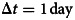 until the last day of available incidence/vaccination data. For dates where data is unavailable, we assume the respective rates are equal to zero.

## Results

We find an estimated nationwide median share of fully susceptible individuals of 5.6% (quartile range [2.5%–8.5%]) until 31 May 2022. This result is, however, biased towards higher values due to a larger share of unaffected infants (36.3% [18.8%–49.8%]) and children (16.7% [4.2%–28.6%]). For age groups that are associated with a higher probability of severe disease [30], we find a lower relative frequency of 3.8% [1.6%–5.9%] (adults), and 2.1% [1.0%–3.4%] (elderly), which is of the same order as the values for adolescents (1.7% [0.1%–5.2%]).

**Fig. 2.**
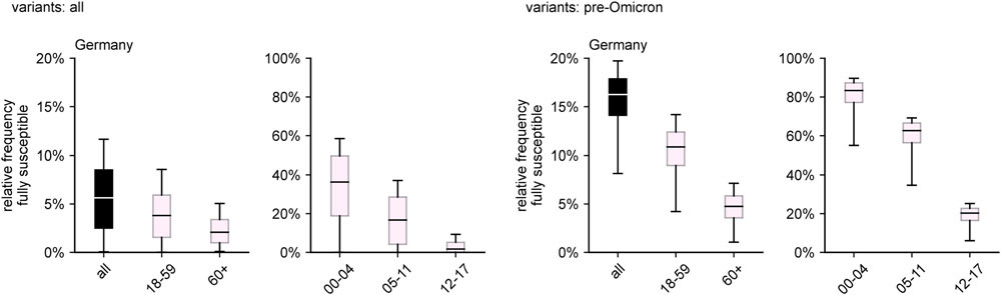
Estimated nationwide relative frequency of fully susceptible individuals by age group, considering vaccinations and infections that took place up to and including May 2022. Boxes represent the area between quartiles *Q*_1_, *Q*_3_ and whiskers the 2.5th and 97.5th percentiles, respectively, the median is shown as a horizontal line. (Left) Considering infections with any variant. (Right) Considering infections with any variant other than Omicron and its sublineages.

These values are achieved largely due to the respective Omicron waves in early 2022. Ignoring infections with the Omicron variant, the nationwide age-independent share of fully susceptibles increases to 16.3% [14.1%–17.9%], i.e. Omicron infections are expected to have caused a reduction in fully susceptible individuals on the order of 10 percentage points up to June 2022, although this number differs by age group. While the change in relative frequency of fully susceptibles in the ‘adult’ and ‘elderly’ age groups was only about a few percentage points (median decreases from 10.8% to 3.8% and from 4.8% to 2.1%, respectively), the three youngest age groups were affected much more strongly, with median values of fully susceptible individuals dropping from 83.3% to 36.3%, from 62.6% to 16.7% and from 20.3% to 1.7% with increasing age (cf. Fig. 2). If all variants are considered, the median share of fully susceptible ‘adults’ and ‘elderly’ barely differs (absolute difference of 1.7% points), likely due to a larger fraction of Omicron-recovered ‘adults’ (Fig. 2).

**Fig. 3.**
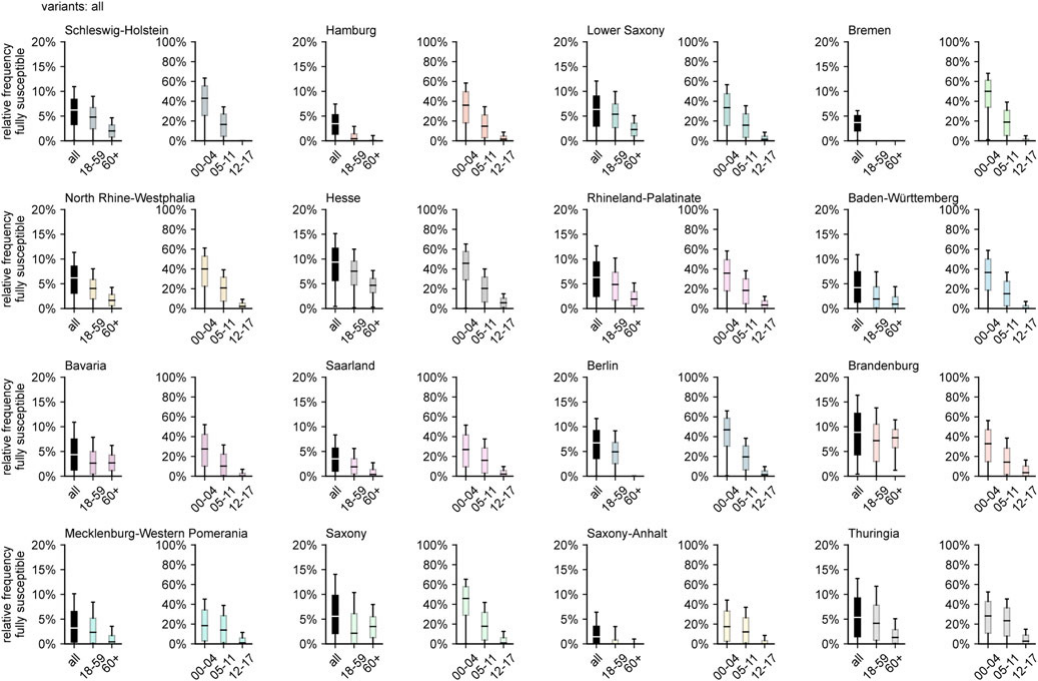
Estimated relative frequency of fully susceptible individuals by age group and region considering infections with any variant and vaccinations up to and including the Omicron wave (as of 31 May 2022).

Although the relative frequency of fully susceptibles varies between federal states, certain commonalities are still shared. In all states, the frequency of fully susceptible individuals decreases with age, with a strong dependence on age for children. For ages 12–17, the frequency reaches values on the same order as those of the age groups ‘adults’ and ‘elderly’ (Fig. 3). Apart from the fact that adult and elderly age groups achieve relative frequencies of fully susceptible individuals below 10%, there are no other common patterns that stand out across all states regarding these age groups. In general, these age groups show overlapping quartile intervals, with the exception of Hamburg and Bremen, where ‘adults’ show a comparatively lower relative frequency (Fig. 3). In fact, in Bremen virtually no one aged 18 and above is expected to not have been in contact with either virus or vaccine, according to the estimations.

**Fig. 4.**
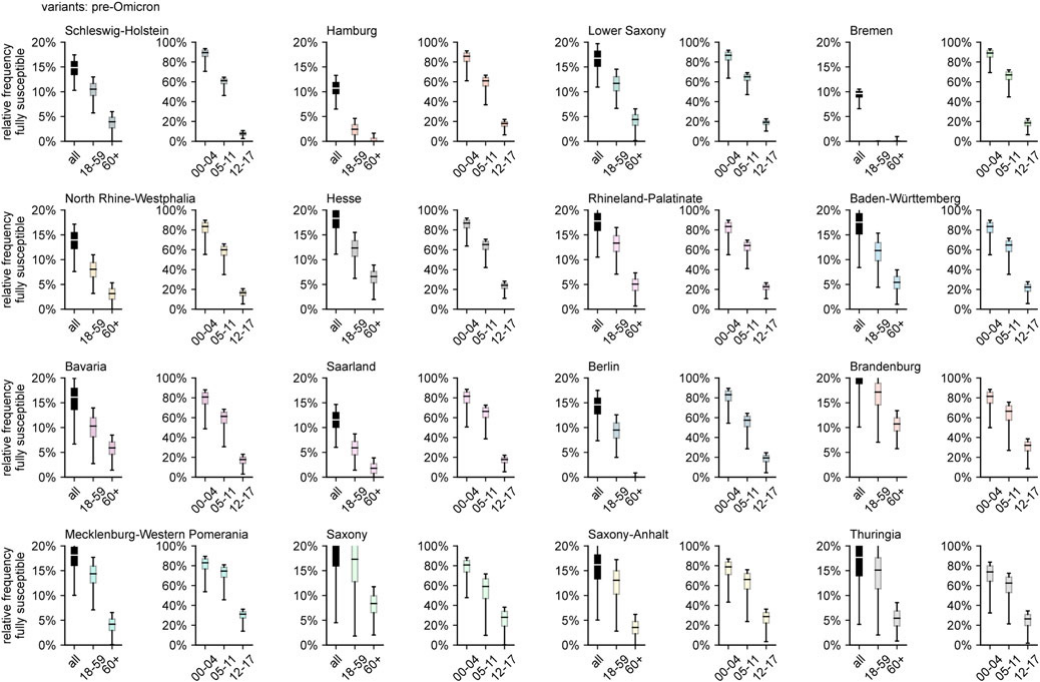
Estimated relative frequency of fully susceptible individuals by age group and region, disregarding infections with Omicron and its sublineages, based on data available up to and including May 2022.

In general, the above observations hold for the pre-Omicron analysis as well, except for the fact that, in the majority of states, the number of adults that were still unaffected decreased dramatically during the Omicron wave due to the large number of infections caused by the variant (comparing Figs 3 and 4). When excluding Omicron infections, the relative frequency of fully susceptibles differs across states on the order of about 10 percentage points, with Brandenburg and Bremen as the states with largest (21.7%) and smallest (9.6%) respective median values of fully susceptible individuals (Fig. 4). Including infections with Omicron, the median range between states is reduced to a difference of 7.9% points (median of 9.4% in Hesse and 1.4% in Saxony-Anhalt).

Our results are robust against changes in assumed eligibility time *τ* and recovered immunity *r*, varying by a few percentage points in the nationwide average for all ages. For the most at-risk age groups, i.e. adults and the elderly, these results vary even less, indicating that the influence of these parameters decreases with age (see SM, Sec. ‘Methods’ and Supplementary Fig. S3).

Regarding the detailed distribution of individuals by vaccination/infection status, we find that the largest single compartment of the model population is the group of people that has received a booster vaccination and has never been in contact with the virus (see SM Sec. ‘Methods’ and Supplementary Fig. S2), with unvaccinated recovereds comprising the second largest group. When first excluding, then including Omicron infections, both the number of non-infected vaccinateds and non-infected booster vaccinateds decreases by about 10 percentage points, demonstrating the relative efficacy of the booster vaccination against infections with the Omicron variant. The prevalence of compartments that count infected individuals decreases with the number of (breakthrough) infections per individual, which is unsurprising given that the model probability to become infected decreases exponentially with every new infection. Note that our model cannot, however, track the number of reinfections per individual between achieving the different vaccination statuses.

As under-ascertainment is expected to be larger for infants than for other age groups, we scaled the respective under-ascertainment ratio to always assume twice the value of other age groups. Because most children below 5 years of age will remain unvaccinated as per official recommendations, only infections reduce the number of fully susceptible individuals, and, therefore, the under-ascertainment ratio has a large influence (see SM, Sec. ‘Methods’ and Supplementary Fig. S4). With the degree of under-ascertainment in this age group comparatively unclear, the results must be considered relatively uncertain for this age group.

## Conclusion & discussion

As the pandemic progresses, a central quantity that will determine the upcoming dynamics is the population-wide susceptibility against infection with known or future variants of SARS-CoV-2. While protection from infection, either derived from vaccination or natural infection, wanes over time and depends on the circulating virus variant, an estimation of the respective subpopulation sizes of people that suffered from one or more infections or were vaccinated/boostered gives valuable information about the size of the population that is still fully susceptible to infection, because these individuals are more prone to infection and severe disease as compared to vaccinated or recovered individuals, given that future variants do not fully escape this immunity.

Here, we found that in Germany, a nationwide single-digit percentage of individuals have not been in contact with either a variant of SARS-CoV-2 or a vaccine against them up to June 2022, yet these results vary between regions and age groups. Despite the high number of reported infections in infants and children, a considerably high percentage of these age groups might still have been fully susceptible to infection after this time. This may become problematic if a variant emerges that causes more severe disease in these age groups than previous variants. Yet, we cannot rule out the possibility that we underestimated the extent of under-ascertainment in these age groups, as the factors we used where informed by seroprevalence studies based on blood samples donated by adults (ages 18–74), while it has been reported that under-ascertainment ratios can assume values ranging from 2 up to 6 or 8 for children [31–33].

In comparison, the age groups of adults and elderly showed a relatively low share of fully susceptible individuals, considering infections with all variants on the order of 5%. Only considering infections with pre-Omicron variants, however, around 9.0%–12.4% of the adult population and 3.6%–5.8% of the elderly population were expected to still be at risk of infection with variants that have a higher probability of causing severe disease than Omicron, potentially causing large outbreaks that could put high pressure on the public health system once again (with these numbers representing quartile ranges).

Across all ages and regions, we found that about 6% of the German population were expected to have not been in contact with either virus or vaccine. While at the time of analysis, no seroprevalence data that extended into the Omicron waves were available [12], recently an interim analysis was published in a repository [34], based on blood samples that were taken between May and September 2022. The report concluded that 95% of the participants showed antibodies against the S-antigen, across all ages in Germany. The results are not directly comparable because these samples were taken after our study period ended and, as discussed, antibody concentration in the blood is known to wane over time, but they are in line with our estimates and support the validity of our method.

Our results are subject to a number of limitations and biases. For instance, the reported uncertainties (quartile ranges) are heavily determined by the choice of distribution of 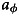. The distribution we chose has a median value of 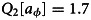, which is slightly lower than what was observed in 2020 [19]. Moreover, the lower distribution bound of 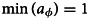 might be rather low, as such a value would mean that every infection has been reported, which is unlikely. Hence, at least the upper percentiles we report for *S*_∞_ might be overestimations. Furthermore, we assume the same distribution of under-ascertainment ratios for all German states, which might not reflect potential heterogeneities in local ascertainment particularly well.

Regarding modelling choices for the eligibility time, a short average duration after infection to be eligible for vaccination leads to larger proportions of vaccine-eligible people and, hence, to a higher overlap between the vaccinated and recovered subpopulations, thus increasing the estimated number of fully susceptibles. While we chose a comparably low value of 90 days for this parameter, lower values cannot be ruled out. However, (i) the value we chose lies below the official recommendation and (ii) changes in this parameter are not expected to change our results drastically, as was shown in a sensitivity analysis.

Likewise, shorter durations of eligibility for reinfection and lower values of long-term immunity of recovered individuals increase the likelihood that a reported infection of an unvaccinated individual was, in fact, a reinfection event, thus leading to higher values of fully susceptible individuals over all. As above, our results are robust towards variations in these parameters.

Regarding results on a regional level, reported vaccinations and infections might be skewed regionally when a large number of people live in one state but traverse to others to seek medical help. These considerations might explain the extreme results observed for Hamburg and Bremen, which are city states enclosed by others.

The last German census took place in 2011 and population sizes per age group and region have been imputed for the year 2020 based on this data, thus potentially being subject to over- or under-counting. Uncertainties in population size may introduce systematic errors on the order of a few percentage points in relative frequencies. When such a relative frequency reaches low values, these absolute errors on the order of a few percentage points can lead to high relative errors in the results.

Considering incidence rates, we imputed the total number of unvaccinated cases per day from cases with undetermined vaccination status by assigning them the ‘unvaccinated’ status with probability proportional to the share of unvaccinated cases in the set of cases with determined status. This procedure can introduce systematic errors when the ascertainment of vaccination status is biased towards any of the vaccination states, which may occur, for instance, when the probability of status ascertainment increases with severity of disease. In this case, people with breakthrough infections may be less likely to have their vaccination status reported in the reporting system, which would mean that we overestimated the number of unvaccinated cases per day, introducing a bias towards lower values of the share of fully susceptible individuals.

For analyses regarding infections with variants prior to Omicron, we relied on the nationwide share of Omicron sequences, multiplying all incidence rates (regardless of region, age or vaccine status) with this function. Since vaccines assume different efficacies against infection with different variants and will likely vary across ages and regions, this assumption is expected to introduce strong bias on a fine-grained population level, which may be expected to decrease when values are aggregated over regions or ages.

Our results cannot be used to predict the future course of the pandemic directly. In fact, since SARS-CoV-2 lacks phenotypical stability and neither infection nor vaccination elicits full long-term protective immunity, especially with respect to the prevention of infection and transmission, classical herd immunity is unlikely to be reached for COVID-19 [35]. In several studies hybrid immunity resulting from infection-acquired immunity boosted with vaccination conferred the strongest or longer-lasting protection, respectively [36, 37]. Similarly, Omicron breakthrough infections in previously vaccinated individuals have been shown to drive cross-variant neutralisation and memory B cell formation [38], suggesting that a combination of both, natural infection and vaccination, will have more impact on the future COVID-19 epidemiology than one of the events alone.

To sum up, our study shows that, presumably, only a small part of the German population has not yet been in contact with either a variant of SARS-CoV-2 or a respective vaccine against the disease they cause, up to and including May 2022. We show important proportions of fully susceptible elderly, who on average, by their age and age-associated morbidities, have a disproportionately elevated risk of severe disease. These shares differ by region and could motivate regionally targeted protection measures at the time of writing or in case of future outbreaks.

While the immunisation campaign was successful in spring and summer 2021, in particular reaching a large proportion of vulnerable people, it thereafter had difficulties to completely close immunity gaps with vaccinations, albeit enhancing the protection of a large proportion of already vaccinated people with a large booster vaccination campaign by the end of 2021. Our results show that the Omicron wave had a high impact on naturally closing the aforementioned gaps. As mentioned above, however, having been in contact with a variant of SARS-CoV-2 is not a robust equivalent of immunity and may range from mild infection followed by rapid waning of antibodies and a highly uncertain degree of immunity, to a fully vaccinated status including a booster and a breakthrough infection, which confers a more long-lasting and robust degree of protection against severe disease. At the lower end of this spectrum of presumed immunity, our analyses show that up to June 2022, one in six persons was never vaccinated but infected once or more, in the majority of cases with Omicron. This group faces higher uncertainties for later infection waves since protection against severe disease is expected to be more short-lived and too narrowly targeted to this variant.

## Data Availability

The code and results of the simulations can be obtained from Zenodo [39].

## Supplementary material

For supplementary material accompanying this paper visit http://doi.org/10.1017/S0950268823000195. click here to view supplementary material
