# Supplementary material for: Estimating the share of SARS-CoV-2-immunologically naïve individuals in Germany up to June 2022
Source: Epidemiol Infect. 2023 Feb 15;151:e38. doi: 10.1017/S0950268823000195 (PMC10028997; doi:10.1017/S0950268823000195)
Supplement: Supplementary file 1 [file S0950268823000195sup001.pdf]

Supplementary Material:
Estimating the share of SARS-CoV-2-immunologically naïve individuals in Germany up to June 2022

Benjamin F. Maier,[1,2,3,*] Annika H. Rose,[1,4] Angelique Burdinski,[1,4]
Pascal Klamser,[1,4] Hannelore Neuhauser,[1] Ole Wichmann,[1]
Lars Schaade,[1] Lothar H. Wieler,[1] and Dirk Brockmann[1,4]

[1]*Robert Koch Institute, Berlin, Germany*
[2]*DTU Compute, Technical University of Denmark, Kongens Lyngby, Denmark*
[3]*Copenhagen Center for Social Data Science, University of Copenhagen, Copenhagen, Denmark*
[4]*Institute for Theoretical Biology and Integrated Research Institute for the Life-Sciences, Humboldt University of Berlin, Berlin, Germany*

(Dated: February 6, 2023)

I. MULTI-STATE MODEL

A. Model formulation

We partition the population into $n_G = 16$ regions corresponding to the German states and $n_A = 5$ age groups corresponding to ages "00-04" (infants), "05-11" (children), "12-17" (adolescents), "18-59" (adults), "60+" (elderly), chosen in accordance with the population structure of publicly available vaccination data [1]. Consequently, for any region- and age-specific compartment $X_{A,G}$, the nation-wide value is given as

$$X_A = \sum_{G=1}^{n_G} X_{A,G}, \tag{S1}$$

the corresponding value for all ages is given as

$$X_G = \sum_{A=1}^{n_A} X_{A,G}, \tag{S2}$$

and the total value is

$$X_{\mathrm{tot}} = \sum_{A=1}^{n_A} \sum_{G=1}^{n_G} X_{A,G}. \tag{S3}$$

Because in the further analysis, none of the subpopulations are interacting, we will omit the region- and age-determining subscripts for simplicity.

For any population of size N, we are first and foremost interested in the number of susceptible individuals S, i.e. the number of individuals that have never been in contact with neither a variant of SARS-CoV-2, nor a vaccine against it. We assume that previous to the pandemic, no individual has had contact with any variant of SARS-CoV-2 or a vaccine against them, i.e. $S(t = 0) = N$. These susceptibles can then either (i) become infected (changing their status to I) or (ii) vaccinated (changing their status to V). The number of individuals changing their status per day is estimated from official data [1, 2], defining the number of reported newly infected unvaccinated individuals per day as $\phi_S(t)$ and the number of newly vaccinated individuals per day as $\beta_S(t)$. We obtain these rates on a calendar-week basis in order to remove weekly modulations. Because the vaccination status of new infections is unknown for a considerable amount of people, we impute ϕ_S from incomplete incidence data in a procedure outlined further below. The rates are to be interpreted in

* bfmaier@physik.hu-berlin.de

a way such that

$$M_S = \int\limits_0^{t_{\max}} dt\, \beta_S(t), \qquad \text{and} \tag{S4}$$

$$F_S = \int\limits_0^{t_{\max}} dt\, \phi_S(t) \tag{S5}$$

give the cumulative number of vaccinated individuals and the cumulative number of reported infections of unvaccinated individuals, respectively, both up to time $t_{\max}$.

At any time t, the number of individuals eligible to receive a vaccine is proportional to (a) the number of susceptible individuals and (b) the number of recovered individuals. We assume that infected individuals become eligible for vaccination after an average amount of time τ passes. Hence, after obtaining an infection, we assume that individuals change their status with rate $1/\tau$ to become eligible (status Y). Then, the probability for a person that becomes vaccinated at time t to be of status S is given as $p_{V,S} = S/(S+Y)$ and for status Y as $p_{V,I} = Y/(S+Y)$. These equations are implicitly based on the assumptions that recovered individuals share the same vaccination intention as susceptible individuals, which is supported by representative survey studies [3]. Consequently, the vaccination transition rate for both susceptibles and eligible recovereds to receive vaccination status is given as

$$\tilde{\beta}_S = \frac{a_\beta \beta_S}{S+Y}. \tag{S6}$$

Here, we further introduced the under-ascertainment ratio of vaccinations a_β. The corresponding transition processes are

$$S \xrightarrow{\tilde{\beta}_S} V \tag{S7}$$

$$Y \xrightarrow{\tilde{\beta}_S} C_{IV} \tag{S8}$$

where C_{IV} represents the compartment counting individuals who became infected at least once before receiving a vaccination. The reaction

$$I \xrightarrow{1/\tau} Y \tag{S9}$$

represents the process of recovered individuals becoming eligible for vaccination.

Similarly, the number of individuals eligible to transition to status “unvaccinated infected” is proportional to (a) the number of susceptible individuals and (b) the number of recovered individuals that are eligible for reinfection. We assume that individuals that recently suffered from

an infection are fully immune, but may return to (partial) susceptibility after an average duration of τ, equating this to the average duration it takes to become eligible for vaccination for model parsimony and reasons outlined further below. Because reinfections are not registered in the German reporting system, we have to consider the relative probability for a recovered person to be reinfected by introducing an "immunity parameter" r that represents the relative probability of a recovered person to become infected after time τ since the last infection as compared to a fully susceptible person. Hence, the total number of people eligible to be counted as an infection of an unvaccinated individual at time t is given as $S + (1 - r)Y$, the probability that an unvaccinated person that becomes infected at time t has been infected before is $p_{I,I} = (1 - r)Y/(S + (1 - r)Y)$, and $p_{I,S} = S/(S + (1 - r)Y)$ that they have been fully susceptible. Consequently, the eligibility-corrected vaccination rate is given as

$$\tilde{\phi}_S = \frac{a_\phi \phi_S}{S + (1 - r)Y}. \tag{S10}$$

Here, a_ϕ is the under-ascertainment ratio, accounting for infections that have not been reported. The corresponding transition processes are

$$S \xrightarrow{\tilde{\phi}_S} I \tag{S11}$$

$$Y \xrightarrow{(1-r)\tilde{\phi}_S} I. \tag{S12}$$

Again, Eq. (S9) represents the process of becoming eligible (both for vaccination after infection and reinfection).

Continuing with this line of argumentation, we further consider the adjusted rate of individuals that obtain a breakthrough infection as

$$\tilde{\phi}_V = \frac{\phi_V}{V + (1 - r)C_{VY} + C_{IV} + (1 - r)C_{IVY}}. \tag{S13}$$

Here, C_{VY} are vaccinated individuals that suffered from a breakthrough infection before, and C_{IVY} counts individuals that, after recovery became vaccinated, then suffered from a breakthrough infection again. The respective transition processes are displayed in Fig. S1.

Similarly, the adjusted booster rate

$$\tilde{\beta}_V = \frac{\beta_V}{V + C_{VY} + C_{IV}} \tag{S14}$$

quantifies the rate with which previously vaccinated individuals receive a booster vaccination (processes shown in Fig. S1).

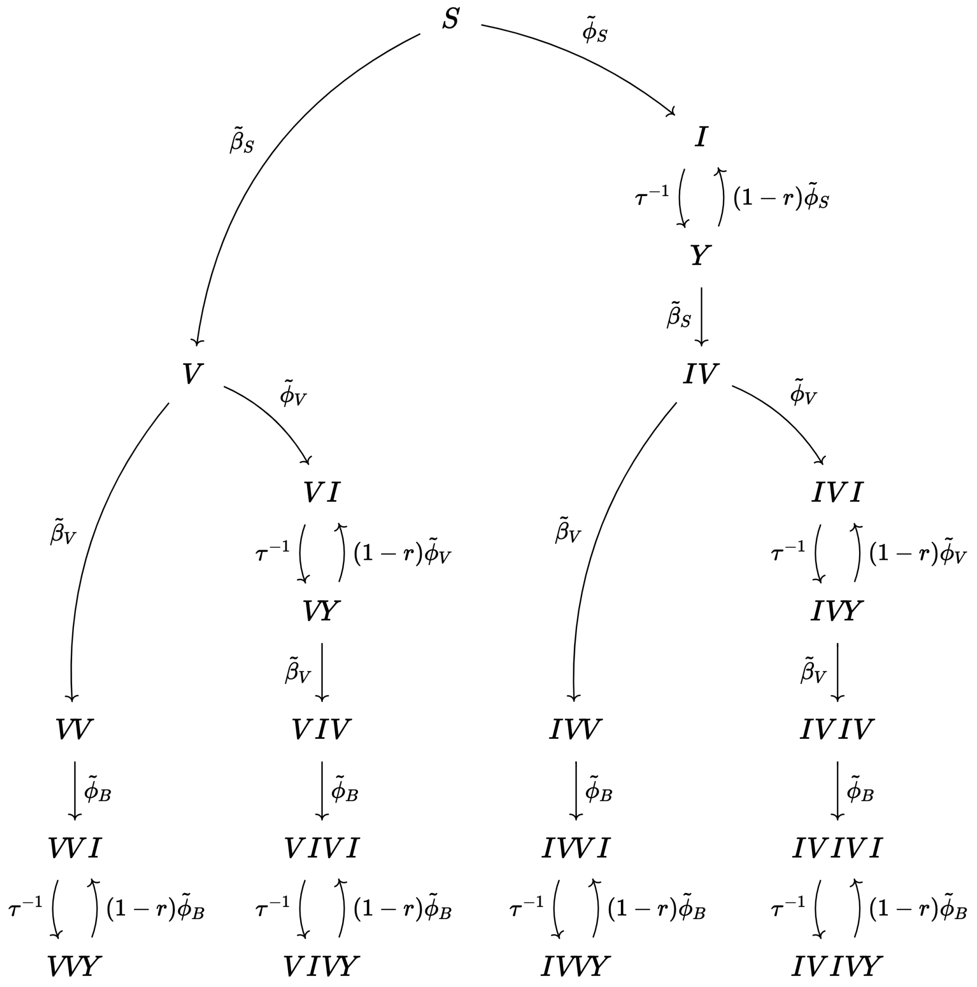

FIG. S1. Vaccination/infection model given by Eqs. (S6)-(S36). Individuals can become infected and recover (compartments ending in I), vaccinated (compartments ending in V), or eligible for reinfection/vaccination after a previous infection after an average duration of τ^{-1} (compartments ending in Y). Initially, all individuals are susceptible (S). Transition rates are determined by data and scaled by assumed under-ascertainment ratios (not shown here). Individuals that are eligible for reinfection are associated with a relative reduction in susceptibility r. The order of I and V in individual statuses represent the order in which infections and vaccinations happened to the respective individuals.

Finally, the adjusted booster breakthrough rate is

$$\tilde{\phi}_B = \frac{\phi_B}{C_{VV} + C_{VIV} + C_{IVV} + C_{IVIV} + (1-r)\left[C_{VVY} + C_{VIVY} + C_{IVVY} + C_{IVIVY}\right]}. \qquad \text{(S15)}$$

For every compartment $C_\bullet$, the order of I and V in the subscript $\bullet$ represents the order in which

infections and vaccinations happened to the individuals counted in the respective compartment.

In total, the model is determined by the following set of ordinary differential equations (ODEs)

$$\partial_t S = -\tilde{\phi}_S S - \tilde{\beta}_S S \tag{S16}$$

$$\partial_t V = \tilde{\phi}_S S - \tilde{\beta}_V V - \tilde{\phi}_V \tag{S17}$$

$$\partial_t I = \tilde{\beta}_S S + (1-r)\tilde{\phi}_S Y - I/\tau \tag{S18}$$

$$\partial_t Y = I/\tau - (1-r)\tilde{\phi}_S Y - \tilde{\beta}_S Y \tag{S19}$$

$$\partial_t C_{IV} = \tilde{\beta}_S Y - \tilde{\beta}_V C_{IV} - \tilde{\phi}_V C_{IV} \tag{S20}$$

$$\partial_t C_{VI} = \tilde{\phi}_V V + (1-r)\tilde{\phi}_V C_{VY} - C_{VI}/\tau \tag{S21}$$

$$\partial_t C_{VY} = C_{VI}/\tau - (1-r)\tilde{\phi}_V C_{VY} - \tilde{\beta}_V C_{VY} \tag{S22}$$

$$\partial_t C_{IVI} = \tilde{\phi}_V C_{IV} + (1-r)\tilde{\phi}_V C_{IVY} - C_{IVI}/\tau \tag{S23}$$

$$\partial_t C_{IVY} = C_{IVI}/\tau - (1-r)\tilde{\phi}_V C_{IVY} - \tilde{\beta}_V C_{IVY} \tag{S24}$$

$$\partial_t C_{VV} = \tilde{\beta}_V V - \tilde{\phi}_B C_{VV} \tag{S25}$$

$$\partial_t C_{VIV} = \tilde{\beta}_V C_{VY} - \tilde{\phi}_B C_{VIV} \tag{S26}$$

$$\partial_t C_{IVV} = \tilde{\beta}_V C_{IV} - \tilde{\phi}_B C_{IVVI} \tag{S27}$$

$$\partial_t C_{IVIV} = \tilde{\beta}_V C_{IVY} - \tilde{\phi}_B C_{IVIV} \tag{S28}$$

$$\partial_t C_{VVI} = \tilde{\beta}_B C_{VV} + (1-r)\tilde{\phi}_B C_{VVY} - C_{VVI}/\tau \tag{S29}$$

$$\partial_t C_{VIVI} = \tilde{\beta}_B C_{VIV} + (1-r)\tilde{\phi}_B C_{VIVY} - C_{VIVI}/\tau \tag{S30}$$

$$\partial_t C_{IVVI} = \tilde{\beta}_B C_{IVVI} + (1-r)\tilde{\phi}_B C_{IVVY} - C_{IVVI}/\tau \tag{S31}$$

$$\partial_t C_{IVIVI} = \tilde{\beta}_B C_{IVIV} + (1-r)\tilde{\phi}_B C_{IVIVY} - C_{IVIVI}/\tau \tag{S32}$$

$$\partial_t C_{VVY} = -(1-r)\tilde{\phi}_B C_{VVY} + C_{VVI}/\tau \tag{S33}$$

$$\partial_t C_{VIVY} = -(1-r)\tilde{\phi}_B C_{VIVY} + C_{VIVI}/\tau \tag{S34}$$

$$\partial_t C_{IVVY} = -(1-r)\tilde{\phi}_B C_{IVVY} + C_{IVVI}/\tau \tag{S35}$$

$$\partial_t C_{IVIVY} = -(1-r)\tilde{\phi}_B C_{IVIVY} + C_{IVIVI}/\tau. \tag{S36}$$

B. Parameters and data

1. Incidence by vaccination status

For each combination of age group and region, we obtain the daily number of reported new cases in unvaccinated $\hat{n}_S(t)$ by "Meldedatum" (date of report), as well as the daily number of reported breakthrough infections $\hat{n}_V(t)$, reported booster breakthrough infections $\hat{n}_B(t)$, as well as the daily number of infections where the vaccination status is unknown $\hat{n}_\varnothing(t)$ from the German reporting system SurvStat [4] In order to assign vaccination statuses to cases where the status is originally unknown, we measure the proportion of infections per status in cases with known status in the last seven days and subsequently obtain the imputed number of daily cases as

$$n_X(t) = \hat{n}_X(t) + \hat{n}_\varnothing(t) \frac{\sum_{t'=t-6\mathrm{d}}^{t} \hat{n}_X(t')}{\sum_{t'=t-6\mathrm{d}}^{t} \left[\hat{n}_S(t') + \hat{n}_V(t') + \hat{n}_B(t')\right]}, \qquad \forall X \in \{S, V, B\}. \tag{S37}$$

This procedure removes weekly modulations for the imputation. It might be biased towards any of the statuses S, V, B due to different probabilities of severe disease by vaccination status and thus of being reported in a system of primarily symptom-based testing. Note that, for no region and age groups there were days for which $\aleph = \sum_{t'=t-6\mathrm{d}}^{t} \left[\hat{n}_S(t') + \hat{n}_V(t') + \hat{n}_B(t')\right] = 0$ and $\hat{n}_\varnothing(t) > 0$, which is why we set $n_X(t) = \hat{n}_X(t) = 0$ on days where $\aleph = 0$. With the above definition, the infection rates are given as

$$\phi_X(t) = \frac{1}{|\mathcal{W}(t)|} \sum_{t' \in \mathcal{W}(t)} n_X(t'), \qquad \forall X \in \{S, V, B\} \tag{S38}$$

where $\mathcal{W}(t)$ is the set of days t' in calendar week of day t meeting $t' < t_{\max}$.

2. Vaccination rates

Similarly, weekly vaccination rates are given as

$$\beta_X(t) = \frac{1}{|\mathcal{W}(t)|} \sum_{t' \in \mathcal{W}(t)} \hat{v}_X(t'), \qquad \forall X \in \{S, V\} \tag{S39}$$

with $\hat{v}_S(t)$ and $\hat{v}_V(t)$ being the number of new vaccinations (new booster vaccinations, respectively) on day t. We define "new vaccinations" as entries in the data provided in ref. [1] that have an "Impfschutz"-field value of "2", and as "new booster vaccinations" as entries that have an "Impfschutz"-field value of "3", ignoring single-shot vaccinations with value "1" (in the data,

confirmed recovered individuals that received a single vector- or mRNA-vaccine dose are counted as being fully vaccinated with an "Impfschutz"-field value of "2"). The share of the population that received only one dose of an mRNA or the Vaxzevria vaccine is expected to be on the order of 1% of the German population up to and including May 2022 [1]. In the model, the infection of these individuals follows the same dynamics as the infection of fully susceptible individuals. Hence, ignoring this vaccination state will barely affect the results.

Note that we ignore the small number of vaccinations associated with the region "Bund" (region id "17").

3. *Under-ascertainment*

Based on seroprevalence data collected over the first waves in Germany, a nation-wide under-ascertainment ratio of $a_\phi \approx 2$ was found, with regional variations that went up to a factor of $a_\phi \approx 5$ in regions of large outbreaks [5, 6]. In absence of more fine-grained and temporally resolved estimations, we assume an under-ascertainment of $a_\phi = 1 + \hat{a}_\phi$ with $\hat{a}_\phi$ being a Gamma-distributed random variable such that $\langle a_\phi \rangle = 2$ and $\mathrm{Std}[a_\phi] = 1$.

It has further been reported that there might be low under-ascertainment in vaccinations [7]. We assume an under-ascertainment of $a_\beta = 1 + \hat{a}_\beta$ with $\hat{a}_\beta$ being a Gamma-distributed random variable such that $\langle a_\beta \rangle = 1.03$ and $\mathrm{Std}[a_\beta] = 0.02$.

Infants are less likely to display symptoms when infected and are not subject to the strict testing strategies applied in schools [8]. A lower ascertainment in this age group is, therefore, a plausible assumption. We hence assume double the value of the under-ascertainment ratio for this age group.

4. *Eligibility time and immunity of recovered individuals*

We assume an average eligibility time of $\tau = 90$d for vaccination after infection or reinfection. Regarding reinfection, this is a reasonable time scale, as it is of the order of the mean duration neutralising antibodies can be found after an infection. For vaccinations, the official assumption for receiving a vaccine after infection has been 3–6 months. In non-representative survey data, it was found that participants generally followed these recommendations, but with a large number of participants waiting less and became vaccinated about 3 months after a confirmed infection. While the cohort of this study is assumed to be composed of highly compliant individuals, the

average time to receive a vaccination is also lowered assuming a large number of asymptomatic infections, where the date of the infection might be unknown to recovered individuals themselves. Note, however, that we test the influence of this parameter on our results in a sensitivity analysis (see App. II).

We recognize that recovered individuals might still have a lowered susceptibility for reinfection even after transitioning to the eligibility state. The “recovered immunity” parameter r quantifies the relative efficacy against reinfection. For the Alpha variant, this efficacy was observed to be lower than the vaccine efficacy against infection by mRNA- or vector-vaccines [9]. but of similar order as the vaccine efficacy against Infection with Delta, taking on values of $r \approx 0.65$ for both. As Omicron is considered to be a variant with partial immune escape, we set a lower default value of $r = 1/2$ for all variants, testing $r = 0$ (no protection against reinfection) and $r = 1$ (full immunity) in sensitivity analyses.

5. *Variant share*

For analyses disregarding infections with Omicron, we obtained sequences that were sampled randomly nation-wide and independent of age [10]. For each calendar week w we obtained the total number $m(w)$ of randomly sampled sequences with date of extraction t that lie in w. We further aggregated the number $m_o(w)$ of randomly sampled sequences that the software framework “scorpio” identified as “Omicron” or “Probable Omicron”. Then, the share of Omicron on day t is given as

$$\sigma(t) = \begin{cases} 0, & t < \text{Aug 1, 2021} \\ 1, & w(t) > w_{\max} \\ m_o(w(t))/m(w(t)) & \text{otherwise,} \end{cases} \tag{S40}$$

with $w_{\max}$ being the last week for which data was available.

For analyses labeled “pre-Omicron” we analyzed the model with all incidence rates being scaled as $\phi_{\bullet,\text{pre-Omicron}}(t) = \phi_\bullet(t)[1 - \sigma(t)]$.

6. *Simulations*

We draw 1,000 pairs of (a_ϕ, a_β) as described above and assume those under-ascertainment ratios to be constant across all respective ages and regions (bar infants, whose under-ascertainment

ratio is set as $a_{\phi,\text{infants}} = \omega a_\phi$ with $\omega = 2$ to account for the fact that under-ascertainment is expected to be higher in this age group, as already discussed above). Then, Eqs. (S16)–(S36) are integrated with Euler's method using a time step of Δt = 1d, starting on Jan 6, 2020 until May 31, 2022. We then obtain the final state of the compartments, and additionally aggregated states as

$$C_{I^*} = I + Y \tag{S41}$$

$$C_{VI^*} = C_{VI} + C_{VY} \tag{S42}$$

$$C_{IVI^*} = C_{IVI} + C_{IVY} \tag{S43}$$

$$C_{VVI^*} = C_{VVI} + C_{VVY} \tag{S44}$$

$$C_{VIVI^*} = C_{VIVI} + C_{VIVY} \tag{S45}$$

$$C_{IVVI^*} = C_{IVVI} + C_{IVVY} \tag{S46}$$

$$C_{IVIVI^*} = C_{IVIVI} + C_{IVIVY} \tag{S47}$$

$$C_{0V1I} = I + Y \tag{S48}$$

$$C_{1V0I} = V \tag{S49}$$

$$C_{1V1I} = C_{IV} + C_{VI} + C_{VY} \tag{S50}$$

$$C_{1V2I} = C_{IVI} + C_{IVY} \tag{S51}$$

$$C_{2V0I} = C_{VV} \tag{S52}$$

$$C_{2V1I} = C_{VIV} + C_{IVV} + C_{VVI} + C_{VVY} \tag{S53}$$

$$C_{2V2I} = C_{IVIV} + C_{VIVI} + C_{IVVI} + C_{VIVY} + C_{IVVY} \tag{S54}$$

$$C_{2V3I} = C_{IVIVI} + C_{IVIVY} \tag{S55}$$

$$C_{1V} = V + C_{IV} + C_{VI} + C_{IVI} + C_{VY} + C_{IVY} \tag{S56}$$

$$C_{2V} = C_{VV} + C_{VIV} + C_{IVV} + C_{IVIV} + C_{VVI} + C_{VIVI} + \tag{S57}$$

$$+ C_{IVVI} + C_{IVIVI} + C_{VVY} + C_{VIVY} + C_{IVVY} + C_{IVIVY} \tag{S58}$$

$$C_{1I} = I + Y + C_{IV} + C_{VI} + C_{VY} + C_{VIV} + C_{IVV} + C_{VVI} + C_{VVY} \tag{S59}$$

$$C_{2I} = C_{IVI} + C_{IVY} + C_{IVIV} + C_{VIVI} + C_{IVVI} + C_{VIVY} + C_{IVVY} \tag{S60}$$

$$C_{3I} = C_{IVIVI} + C_{IVIVY}. \tag{S61}$$

These states combine compartments that have certain commonalities, e.g. compartments C_{nVmI} is the number of individuals that were vaccinated n times and infected m times (re-infections excluded), C_{nV} is the number of individuals that were vaccinated n times, and C_{mI} is the number

of individuals that were infected m times (re-infections excluded, which means that if an individual was infected $m = 3$ times, they must have been infected before, between, and after the respective inoculations.

We test how robust our results are if per region and age group, individual pairs (a_ϕ, a_β) were drawn from their respective distribution, i.e. assuming heterogeneous under-ascertainment in ages and regions per simulation run, which could potentially change the width of the distribution of respective aggregated values, finding that it does not have a substantial effect.

The results of these simulations can be obtained from Zenodo [11].

II. SENSITIVITY AND OTHER ANALYSES

Nation-wide results for all compartments as well as Eqs. ((S41)–(S61)) can be found in Fig. S2. The compartment with the largest share of the population is C_{VV}, i.e. boostered and never infected, assuming a value of 41.5% [35.2%–46.0%]. Considering all variants, the second largest value can be found for individuals that have never been vaccinated but infected once or more with C_{I^*} assuming 16.4% [13.4%–19.1%]. This value is considerably lower (5.6% [4.3%–7.5%]) when infections with Omicron are excluded. Likewise, the share of vaccinated, yet non-infected individuals V is estimated to assume 13.6% [12.5%–14.3%] with Omicron infections excluded, but 6.3% [3.8%–8.4%] considering all variants. With Omicron infections excluded, the boostered and non-infected population assumes an estimated size of 55.8% [54.0%–57.1%], demonstrating the increased efficacy of the booster vaccination against infection with Omicron as compared to individuals who only finished the first vaccination series.

Regarding the influence of eligibility time, higher values lead to a lower probability of reinfections and vaccinations of recovereds during the most active period of the vaccination campaign, implying the estimated number of fully susceptible individuals decreases with increasing τ. Likewise, the assumed immunity of recovereds r leads to a decreasing value of fully susceptible individuals. The results we reported above lie central within the range of results for extreme value pairs of $\tau = 30$d, $r = 0$ (low), as well as $\tau = 150$d, $r = 1$ (high). For instance for all ages, the results vary between median values of 8.3% (low) and 3.1% (high) with our reported result in the main text ($\tau = 90$d, $r = 0.5$) being equal to 5.6%. The influence of these parameters are higher for the younger population with a "low"-to-"high" variation leading to respective median ranges of 44.8% to 27.5% (infants), 27.0% to 5.7% (children), and 6.3% to 0.0% (adolescents). In the older

population, the influence of these parameters is rather small, leading to median ranges of 5.9% to 1.8% (adults) and 2.9% to 1.1% (elderly). These results are displayed in Fig. S3.

In the main text, we assumed that the relative under-ascertainment factor in infants assume a value of $a_{\phi,\text{infants}}/a_\phi = \omega = 2$. For $\omega = 1$, fully susceptible infants is higher than what we reported in the main text (see Fig. S4). Since empirical values for ω are difficult to obtain, we are probably underestimating the uncertainty in our results for infants.

III. ADDITIONAL, SOPHISTICATED MODEL

We further want to develop a model that allows waning to be included in the analyses and could therefore potentially be used to estimate seroprevalence in future studies.

We hypothesize that exposure to either the pathogen or a vaccine results in an initial immune response that then decays over a period of time and account for this by introducing intermediate compartments representing different gradations of immunity.

We define as S susceptibles, I infected, V vaccinated, Y breakthroughs from vaccinated V and U as breakthroughs from boostered B. For each compartment X, we consider $n_X + 1$ gradations, i.e. we assume that individuals who reach the status X pass through intermediate compartments in the form of a chain from initial X_0 to final $X_{n,X}$, per transition $X_i \longrightarrow X_{i+1}$ with transition rate $1/\tau_{X,i+1}$. This means that for each individual, each of these transitions is subject to a random delay

$$T_{X,i} \sim \text{Exp}(1/\tau_{X,i+1}) \tag{S62}$$

where $\text{Exp}(\lambda_X)$ is an exponential distribution with mean λ_X^{-1}. This approach allows us to more accurately model both waning of immunity and the timing of vaccination or breakthrough infection. For susceptibles, we set $n_S = 0$, i.e. no transitions and exactly one gradation.

We denote $\hat{X}$ as the total number of individuals in status X that are susceptible to infection. That is, we define

$$\hat{X} = \sum_{i=0}^{n_X} (1 - e_{X,i}) X_i, \tag{S63}$$

where $e_{X,i}$ is the susceptibility reduction of a person in status X_i (due to previous infection or vaccination).

We define $\tilde{X}$ as the total number of individuals in status X who can receive one or the next vaccination. Usually, this is the case after a defined time Θ_X has passed since the last infection or

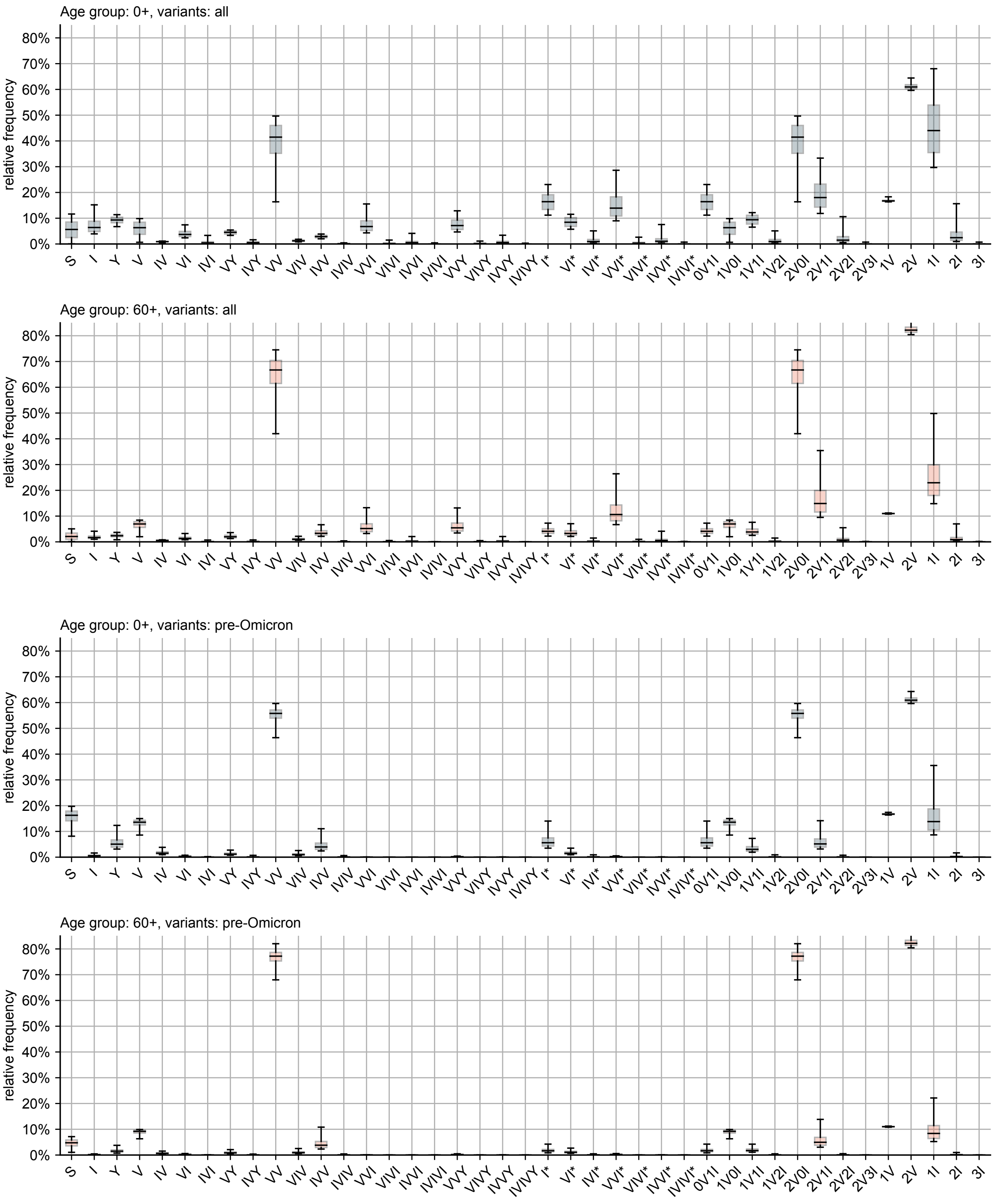

FIG. S2. Relative frequency of all compartments given by vaccination and infection status across Germany, for all age groups and variants as well as for the elderly and pre-Omicron variants. Some compartments shown are aggregates, e.g. labels "nVmI" represent the number of individuals that were vaccinated n times and infected m times (re-infections excluded), labels "nV" give the number of individuals that were vaccinated n times, and labels "mI" are the number of individuals that were infected m times (re-infections excluded), see Eqs. ((S41))-((S61))

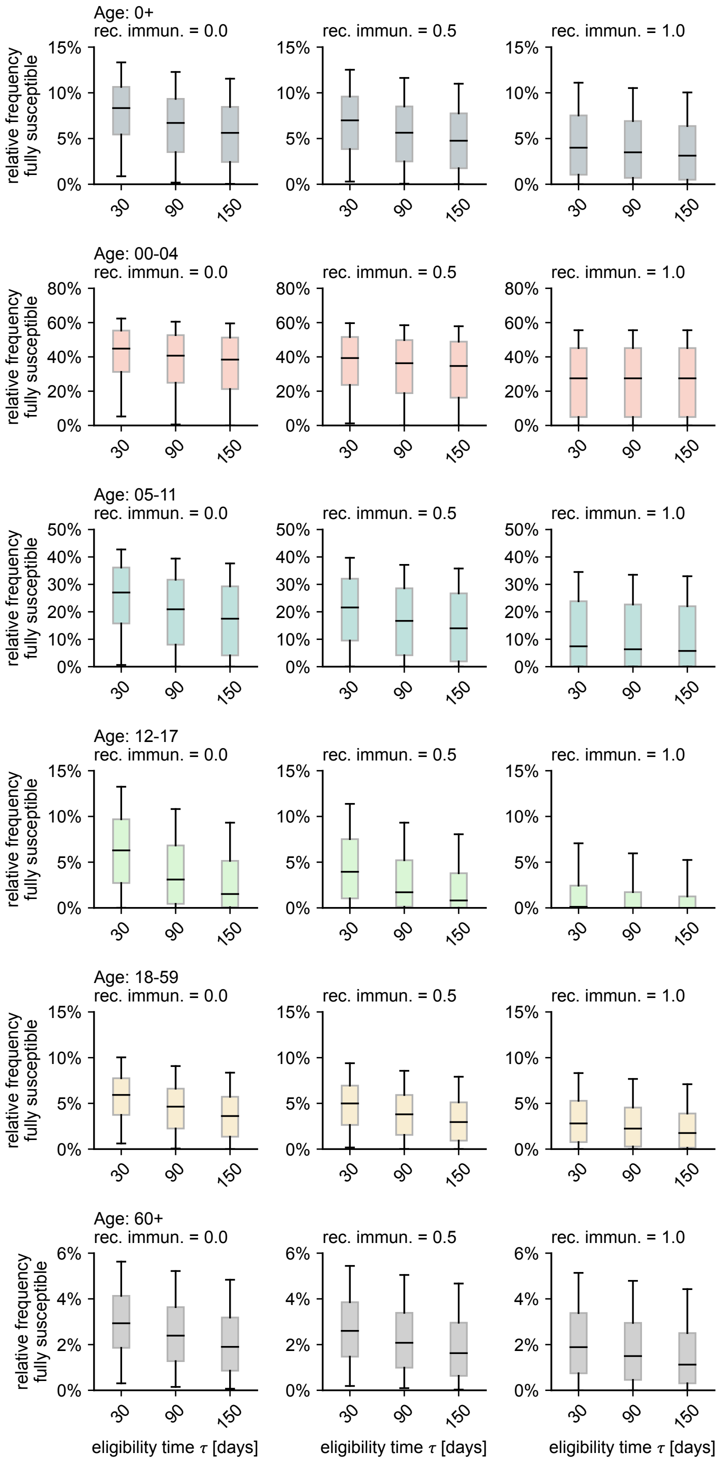

FIG. S3. The influence of the assumed average eligibility duration as well as the long-term immunity of recovered individuals.

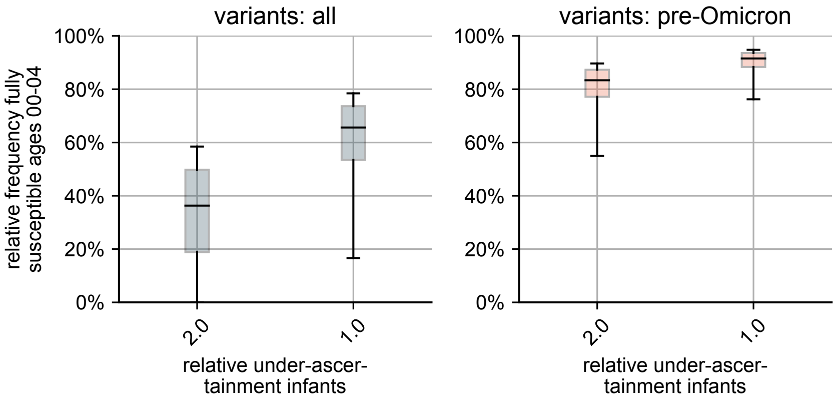

FIG. S4. Influence of relative under-ascertainment for infants. For the main results, we assumed that the relative under-ascertainment factor assumes, for infants, a value of $a_{\phi,\text{infants}}/a_{\phi} = \omega = 2$. For $\omega = 1$, the number of yet fully susceptible infants is higher than what we reported in the main text.

the last receipt of a vaccine dose (comparable to the 'eligibility time' used in the main analyses of this study). The total time it takes for an individual in status X_i to reach status X_{i+1} is given by the random variable

$$Z_{X,i} = \sum_{j=0}^{i} T_{X,j}. \tag{S64}$$

Let $F_{X,i}(z)$ be the cumulative distribution function of the random variable $Z_{X,i}$. Then, the probability $w_{X,i}$ that a given individual in status X_i has been in status X for longer than Θ_X is given by

$$w_{X,i} = P(Z_{X,i} > \Theta_X) = 1 - F_{X,i}(\Theta_X). \tag{S65}$$

We find such

$$\tilde{X} = \sum_{i=0}^{n_X} \left[1 - F_{X,i}(\Theta_X)\right] X_i. \tag{S66}$$

The probabilities $w_{X,i} = 1 - F_{X,i}(\Theta_X)$ are constant and can thus be determined numerically after defining the times $\{\tau_{X,i}\}$ and Θ_X. For susceptibles, let $S = \hat{S} = \tilde{S}$.

Let $\mathcal{I}(X)$ be the compartment to which an individual in status X transitions after infection and $\mathcal{V}(X)$ be the compartment to which an individual in status X transitions after vaccination. We define the following transitions

$$\mathcal{I}(S) = \mathcal{I}(I) = I \tag{S67}$$

$$\mathcal{V}(S) = \mathcal{V}(I) = V, \tag{S68}$$

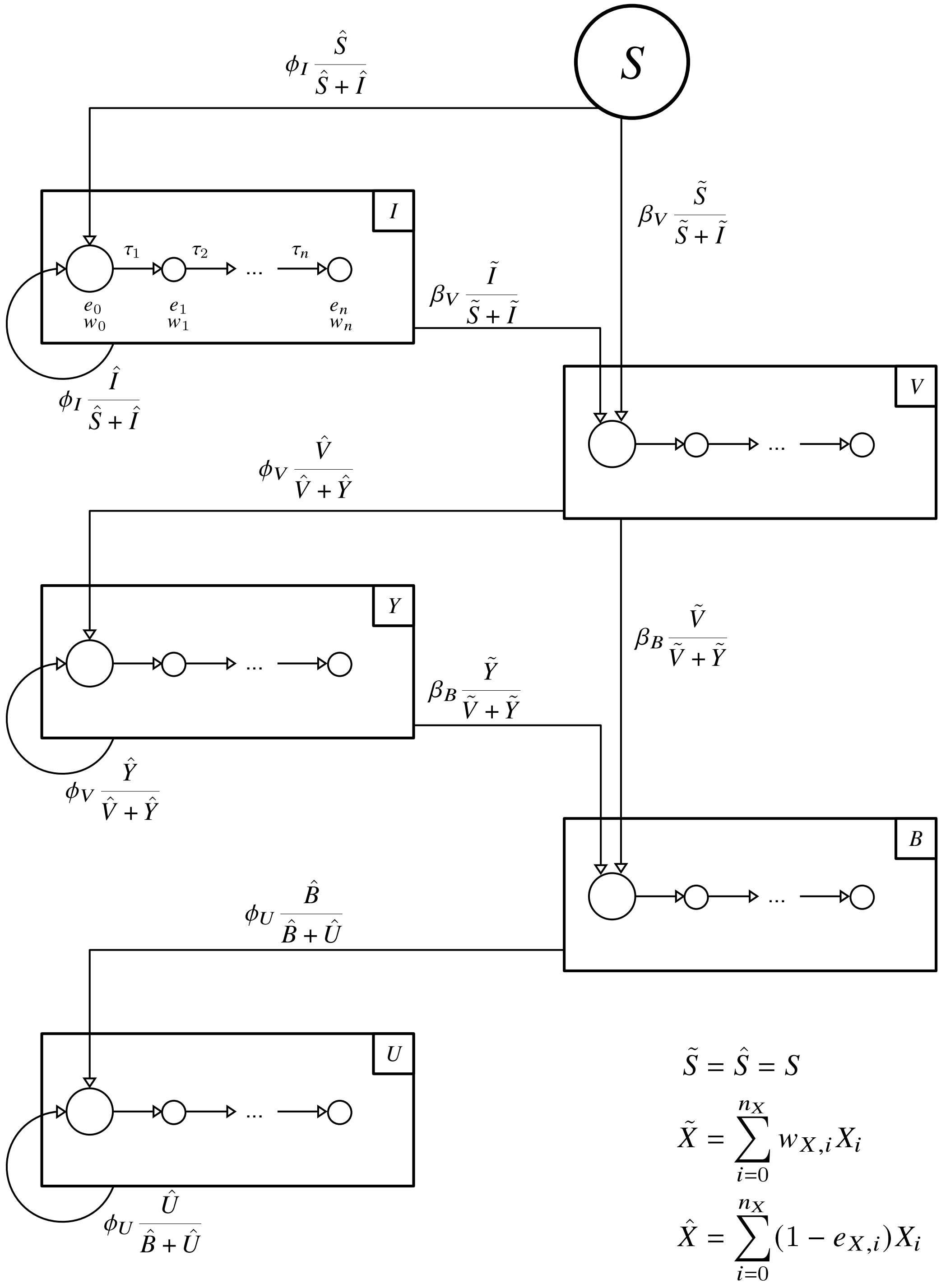

FIG. S5. Detailed model that includes waning.

i.e. susceptibles S who become infected transition to status I and susceptibles who are vaccinated transition to status V. Recovered I who become infected again transition to status I and recovered people who get vaccinated transition to status V. Furthermore,

$$\mathcal{I}(V) = \mathcal{I}(Y) = Y \tag{S69}$$

$$\mathcal{V}(V) = \mathcal{V}(Y) = B, \tag{S70}$$

i.e. vaccinated individuals V who become infected transition to status Y and those vaccinated that receive a third dose transition to status B. Breakthrough-recovereds Y who become reinfected again transition to status Y and breakthrough-recovered individuals who become vaccinated transition to status B. Last,

$$\mathcal{I}(B) = \mathcal{I}(U) = U \tag{S71}$$

$$\mathcal{V}(B) = \mathcal{V}(U) = \varnothing, \tag{S72}$$

i.e. boostered persons B who become infected transition to status U but further vaccination is not provided. Recovered booster vaccinated persons U who become infected again will again transition to status U. The dynamics of all states X_i follows

$$\partial_t X_i = \underbrace{\phi_X \delta_{i,0} - \phi_{\mathcal{I}(X)} \left(1 - e_{X,i}\right) X_i}_{\text{infections}} + \underbrace{\beta_X \delta_{i,0} - \beta_{\mathcal{V}(X)} \left(1 - F_{X,i}(\Theta_X)\right) X_i}_{\text{vaccinations}} + \underbrace{\frac{X_{i-1}}{\tau_{X,i}} - \frac{X_i}{\tau_{X,i+1}}}_{\text{waning}}. \tag{S73}$$

By definition, we have $X_j = 0 \forall j < 0 \wedge j > n_X + 1$, as well as $\phi_\varnothing = 0$ and $\beta_\varnothing = 0$. Furthermore, we set $\beta_S = \beta_I = \beta_Y = \beta_U = 0$ and $\phi_S = \phi_V = \phi_B = 0$, that is, there are no infections ending in vaccination compartments and no vaccinations ending in infection compartments and no transitions ending in S. Additionally, susceptibles are maximally susceptible (i.e. $e_S = 0$) and from $n_S = 0$ follows $w_S = 1$. To ensure the validity of transition terms in intermediate compartments, we additionally define $\tau_{X,j} \neq 0 \forall X, j \leq 0 \wedge j > n_X + 1$.

With regard to under-reporting, we assume that under-ascertainment ratios are already included in the respective rates $\phi_\bullet$ and $\beta_\bullet$.

Finally, the aim of this analysis is to estimate seroprevalence at time t. For each state $X_i \neq S$, we denote by $p_{X,i}$ the probability that antibodies are found in a person in state X_i. Then, the seroprevalence P of the age group/population of consideration is given as

$$P(t) = \sum_{X \neq S} \sum_{i=0}^{n_X} p_{X,i} X_i(t). \tag{S74}$$

The model is illustrated in Fig. S5.

A large number of parameters are required to calibrate the model. For each state $X \in \{I, V, Y, B, U\}$ the number of transitions n_X have to be defined, then n_X mean transition times as well as $n_X + 1$ susceptibility reductions. For compartments I, V, Y and B, eligibility times $\Theta_\bullet$ for receiving a vaccination are to be determined. From reporting data, we obtain the daily number of new infections of unvaccinated $\phi_I(t)$, vaccinated $\phi_V(t)$ and boostered $\phi_U(t)$ individuals. From the vaccination archive, we obtain the daily number of completed initial vaccination series $\beta_V(t)$ and booster vaccinations $\beta_B(t)$. Under-reporting of infections and booster vaccinations must be estimated and accounted for in the respective rates. For each state $X_i \neq S$, the probability $p_{X,i}$ of finding antibodies in a person in state X_i must also be defined.

All these parameters have to be determined for each of the subpopulations (age groups, regions).

---

[1] Robert Koch-Institut, Fachgebiet 33. COVID-19-Impfungen in Deutschland. Zenodo; 2021. Available from: https://doi.org/10.5281/zenodo.5126652.

[2] Robert Koch-Institut. SARS-CoV-2 Infektionen in Deutschland. Zenodo; 2022. Available from: https://doi.org/10.5281/zenodo.5989727.

[3] Betsch C, Korn L, Felgendreff L, Eitze S, Schmid P, Sprengholz P, et al. COSMO – Zusammenfassung und Empfehlungen Wellen 52 und 53. 2021. Available from: https://projekte.uni-erfurt.de/cosmo2020/web/summary/52-53/.

[4] Faensen, D and Krause, G. SurvStat@RKI – a web-based solution to query surveillance data in Germany. Weekly releases (1997–2007) Euro Surveill. 2004;8(22). Available from: https://www.eurosurveillance.org/content/10.2807/esw.08.22.02477-en.

[5] Neuhauser H, Rosario AS, Butschalowsky H, Haller S, Hoebel J, Michel J, et al. Nationally representative results on SARS-CoV-2 seroprevalence and testing in Germany at the end of 2020. Scientific Reports. 2022;12(1):19492. Available from: https://doi.org/10.1038/s41598-022-23821-6.

[6] Neuhauser H, Buttmann-Schweiger N, Ellert U, Fiebig J, Hövener C, Offergeld R, et al. Seroepidemiological studies on SARS-CoV-2 in samples from the general population and blood donors in Germany—Findings up to August 2021. Epidemiologisches Bulletin. 2021;37:3-12. Available from: https://www.rki.de/EN/Content/infections/epidemiology/outbreaks/

COVID-19/EB-37-2021-Art_02_en.pdf.

[7] Robert Koch-Institut. Wöchentlicher Lagebericht des RKI zur Coronavirus-Krankheit-2019 (COVID-19). 2021. Available from: https://www.rki.de/DE/Content/InfAZ/N/Neuartiges_Coronavirus/Situationsberichte/Wochenbericht/Wochenbericht_2021-12-23.pdf?__blob=publicationFile.

[8] Autorengruppe Corona-KiTa-Studie (2021). Quartalsbericht der Corona-KiTa-Studie 6. Quartalsbericht (IV/2021). DJI. 2021. Available from: https://corona-kita-studie.de/quartalsberichte-der-corona-kita-studie.

[9] Office for National Statistics (UK). Coronavirus (COVID-19) Infection Survey Technical Article: Impact of vaccination of testing positive in the UK: October 2021. 2021. Available from: https://www.ons.gov.uk/peoplepopulationandcommunity/healthandsocialcare/conditionsanddiseases/articles/coronaviruscovid19infectionsurveytechnicalarticleimpactofvaccinationontestingposi[illegible]october2021.

[10] Robert Koch-Institut. SARS-CoV-2 Sequenzdaten aus Deutschland. Zenodo; 2022. Available from: https://doi.org/10.5281/zenodo.6381314.

[11] Maier B. serocov: Estimating the distribution of COVID-19-susceptible, -recovered, and -vaccinated individuals in Germany up to June 2022. Zenodo; 2022. Available from: https://doi.org/10.5281/zenodo.7308182.
